# Prognostic role of preoperative inflammatory markers in postoperative patients with colorectal cancer

**DOI:** 10.3389/fonc.2023.1064343

**Published:** 2023-03-29

**Authors:** Zilong Xiao, Xinxin Wang, Xiaoxiao Chen, Jiawei Zhou, Haitao Zhu, Jiangnan Zhang, Wensheng Deng

**Affiliations:** ^1^Department of General Surgery, First Affiliated Hospital of Nanchang University, Nanchang, China; ^2^Laboratory of Digestive Surgery, Nanchang University, Nanchang, China; ^3^Department of Radiation Oncology, The Third Hospital of Nanchang, Nanchang, China; ^4^Department of Intensive Care Unit, Jining Public Health Medical Center, Jining, China

**Keywords:** colorectal cancer, neutrophil-to-lymphocyte ratio, platelet-to-lymphocyte ratio, lymphocyte-to-monocyte ratio, overall survival

## Abstract

**Background:**

Inflammatory response markers are prognostic factors for several cancers, but their role in postoperative colorectal cancer (CRC) is unclear. The purpose was to evaluate the role of preoperative Neutrophil-to-Lymphocyte ratio (NLR), Platelet-to-Lymphocyte-ratio (PLR), and Lymphocyte-to-Monocyte ratio (LMR) in the prognosis of postoperative CRC patients.

**Methods:**

We retrospectively reviewed 448 CRC patients who had undergone surgical resection from December 2015 to December 2017 in our hospital. The plasma NLR, PLR, LMR, CEA, and CA19-9 were collected within 2 weeks before the operation. We recorded the clinical characteristics and survival data by reviewing medical records and phone calls. We analyzed preoperative inflammatory markers and clinical features using Pearson chi-squared tests or Fisher’s tests. Uni- and multivariate Cox regression analyses were performed, and overall survival (OS) was estimated with the Kaplan–Meier method.

**Results:**

High NLR and PLR were associated with worse overall survival in postoperative CRC (HR = 2.140, 95%CI = (1.488-3.078), *P* < 0.001; HR =1.820, 95%CI = (1.271-2.605), *P* = 0.001). High LMR was associated with improved overall survival in postoperative CRC (HR = 0.341, 95%CI = (0.188-0.618), *P* < 0.001). In the multivariate regression analysis, the increase of NLR resulted in an increase in the risk of death (HR = 1.678, 95%CI = (1.114-2.527), *P* = 0.013), and for the LMR, a reduction of the risk of death (HR = 0.480, 95%CI = (0.256 - 0.902), *P* = 0.023). Moreover, TNM stage, CA-199, CEA, nerve or vascular invasion (NVI) and adjuvant chemotherapy after surgery also were associated with worse overall survival in postoperative CRC.

**Conclusion:**

Current evidence indicates that preoperative inflammatory markers NLR, LMR, and PLR are associated with overall survival in postoperative patients with colorectal cancer. NLR is an independent risk factor, and LMR is an independent protective factor in CRC patients after surgery.

## 1 Introduction

Colorectal cancer (CRC) is the third most common malignant tumor globally and the second major cause of cancer death (1). Despite rapid advances in surgical treatment and therapeutic techniques, including chemotherapy, radiotherapy, and immunotherapy, rapid progression and extensive metastasis still led to a low 5-year survival rate of less than 20% (2). It is essential to predict the prognosis of patients for implementing adjuvant chemotherapy or radiotherapy. The TNM stage has become the first reference for the clinical diagnosis and treatment of CRC. In addition, lymph node metastasis directly affects the therapeutic strategies and prognosis of patients with colorectal cancer. Although lymph node metastasis can mostly be diagnosed by computed tomography (CT) and magnetic resonance imaging (MRI), these radiographic examinations sometimes cannot detect micro-metastases. Therefore, preoperative diagnosis of mesenteric lymph node metastasis in patients with CRC is difficult but essential in clinical practice. It is imperative to explore more convenient and inexpensive predictors of lymph node metastasis and prognosis of CRC.

The early diagnosis of CRC and risk evaluation of lymph node metastasis still lack readily available and reliable molecular markers. Inflammation plays a vital role in cancer, which has recently become a research hotspot. Circulating inflammatory biomarkers can influence the postoperative prognosis and outcome of cancer and have become the prognostic markers in various types of cancer patients (3, 4). Systemic inflammation is associated with stage, lymph node metastasis, and long-term prognosis. Tumor cells promote systemic inflammation *in vivo* and alter white blood cell (WBC), and platelet (PLT) counts in peripheral blood (5). These cancer cells release stress-related substances and proinflammatory cytokines, such as tumor necrosis factor α, which aggravate tumor formation and progression (6). The neutrophil-to-lymphocyte ratio (NLR), platelet-to-lymphocyte ratio (PLR), lymphocyte-to-monocyte ratio (LMR), and platelet-to-neutrophil ratio (PNR) of the peripheral blood obtained from a routine blood examination have been implied as indicators of systemic inflammation (7). Recent studies have shown that NLR, PLR, and LMR have essential reference values for the early diagnosis and prognosis of lung, gastric, breast, and liver cancer, as well as the prediction of lymph node metastasis (8–10). However, a relationship between inflammatory biomarkers and the prognosis of CRC has not been completely elucidated.

The present study aimed to investigate the value of circulating systemic inflammatory biomarkers for predicting prognosis in patients with CRC. In this study, we found that preoperative NLR and LMR are independent prognostic factors for patients with CRC. This study facilitates the screening of patients with poor prognoses for individualized treatment, improving the prognosis of patients.

## 2 Patients and methods

A single-institution, retrospective study included 448 patients with stage I to IV CRC who had surgery at the Department of Gastrointestinal Surgery, the First Affiliated Hospital of Nanchang University, between January 2015 and December 2017. The following were the inclusion criteria: (1) patients with stage I-IV CRC who underwent surgical resection, (2) no previous chemotherapy or radiation treatment before surgery, (3) have clear margins at histopathology for the primary tumor site, (4) available preoperative complete blood count, the CEA, and CA19-9 values all within two weeks before surgery and (5) standard adjuvant therapy following surgery. Because there are relatively few cases of preoperative neoadjuvant chemoradiotherapy in our hospital and chemoradiotherapy may affect the inflammatory response, we did not include it. After surgery, stage II patients were mainly single-agent oral capecitabine, and stage III-IV patients were mainly treated with oxaliplatin/irinotecan + capecitabine, and a small number of patients were combined with radiotherapy. The following were the criteria for exclusion: (1) incomplete/inaccurate medical records, (2) histology other than adenocarcinoma, such as neuroendocrine tumors, squamous cell carcinoma, and other types of cancers, (3) presence of hematologic malignancies and disorders that could significantly affect inflammatory markers, and (4) patients diagnosed with previous or concurrent malignancies. Based on our inclusion and exclusion criteria, we included 448 patients in this study and obtained informed consent from all patients. This research was authorized by the ethical committee of the First Affiliated Hospital of Nanchang University.

### 2.1 Follow-up of patients

We regularly followed all postoperative patients every 3 months for 2 years, every 6 months for 3 to 5 years, and every year after 5 years. Follow-up examinations routinely included physical examination and blood laboratory tests, including serum CEA and CA19-9 tests. In most patients, abdominopelvic computed tomography (CT) and chest X-rays or CT were performed within 3-6 months. Other examinations, such as colonoscopy, pelvic magnetic resonance imaging, or positron emission tomography-computed tomography (PET-CT), were performed at the physician’s discretion. Patients were followed until March 30, 2022, or the death of patients.

### 2.2 Blood samples and reference values

Nurses drew blood samples from venous blood within 2 weeks before the surgery date. The blood samples are tested for complete blood count, the CEA, and the CA19-9 value. The reference range is (1.8-6.3) × 10^9^/L for neutrophils count, (125-350) × 10^9^/L for platelets count, (0.1-0.6) × 10^9^/L for monocytes count, and (1.1-3.2) × 10^9^/L for lymphocytes count, the reference range of CEA value is 0-6.5 ng/mL, the reference range of CA19-9 value is 0-27 U/ml. CEA levels ≥6.5 ng/mL were considered positive, and CA19-9 levels of 27 U/mL were considered positive. The NLR and PLR were calculated by dividing the absolute number of neutrophils or platelets by the absolute number of lymphocytes. And the LMR was calculated by dividing the absolute number of lymphocytes by the absolute number of monocytes.

### 2.3 Collection of clinical characteristics

The clinical characteristics of all colorectal cancer patients, including sex, age at diagnosis, body mass index (BMI), clinical pathological stage (stage I-IV), TNM stage (AJCC, version 8), degree of tumor differentiation, tumor primary site (colon, rectum), tumor size (diameter <5cm, ≥5cm), nerve or vascular invasion (NVI), preoperative neutrophil, lymphocyte, and platelet count, preoperative CEA and CA19-9 level, whether adjuvant chemotherapy was given after surgery and survival status (alive/died), were recorded through the review of medical records and a phone call. The overall survival (OS) time was measured from the date of surgery to the date of death from any cause or most recent follow-up. The survival and follow-up data were obtained by collecting outpatient clinical records or directly contacting the patient or their relatives through a phone call from January 1, 2015, to March 30, 2022.

### 2.4 Statistical analysis

The IBM SPSS version 26.0 software for mac and R version 3.6.3 (R Project) was used for data analysis. Statistical significance was set at p<0.05. Categorical variables were compared using the χ^2^ test or Fisher’s test. The receiver operator curve (ROC) was used to analyze the predictive value of prognosis. Univariate and multivariate analyses were performed using Cox regression models. Survival analysis was performed by Kaplan-Meier analysis, and comparison between groups was performed by Log-rank test. The test level was α =0.05, both were two-sided tests, and *P* < 0.05 was considered statistically significant.

## 3 Results

### 3.1 Patient characteristics

A total of 448 patients were finally included in the current study, including 263 (58.7%) males and 185 (41.3%) females. The median follow-up period was 62 months (range 61–64). The mean age was 58.04 ± 11.76 years (range, 21–85). The mean BMI was 22.80 ± 3.29 (range 13.50–34.72). A total of 241 patients (53.8%) had colon cancer, and the remaining 207 patients (46.2%) had rectal cancer. The evaluation of TNM stages revealed that the clinical pathological diagnoses were 260 (58.0%) patients of stage I-II and 188 (42.0%) patients of stage III-IV. As shown in **Table 1**, we found significant differences in tumor site (*P* = 0.003), tumor size (*P* = 0.001) and chemotherapy (*P* = 0.030) between the low and high NLR groups. The remaining clinical parameters, such as sex, age, body mass index (BMI), pathological stage, histologic grade, vascular or nerve invasion (NVI), CEA, and CA19-9, were not different. For PLR, age (*P* = 0.006), BMI (*P* = 0.007), tumor site (*P* < 0.001), tumor size (*P* = 0.005) and chemotherapy (*P* = 0.002) were significant different. For LMR, sex (*P* < 0.001) and tumor site (*P* = 0.045) were different among two groups.

**Table 1:** Characteristics of Patients (n=448).

Characteristic	NLR<2.81	NLR≥2.81	p	PLR<168.24	PLR≥168.24	p	LMR<5.46	LMR≥5.46	p
N	335	113		247	201		347	101	
Sex			0.114			0.101			< 0.001
female	146 (32.6%)	39 (8.7%)		93 (20.8%)	92 (20.5%)		128 (28.6%)	57 (12.7%)	
male	189 (42.2%)	74 (16.5%)		154 (34.4%)	109 (24.3%)		219 (48.9%)	44 (9.8%)	
Age			1.000			0.006			1.000
<60	175 (39.1%)	59 (13.2%)		114 (25.4%)	120 (26.8%)		181 (40.4%)	53 (11.8%)	
≥60	160 (35.7%)	54 (12.1%)		133 (29.7%)	81 (18.1%)		166 (37.1%)	48 (10.7%)	
BMI			0.237			0.007			0.101
<25	243 (54.2%)	89 (19.9%)		170 (37.9%)	162 (36.2%)		264 (58.9%)	68 (15.2%)	
≥25	92 (20.5%)	24 (5.4%)		77 (17.2%)	39 (8.7%)		83 (18.5%)	33 (7.4%)	
Tumor Site			0.003			< 0.001			0.045
Colon	166 (37.1%)	75 (16.7%)		114 (25.4%)	127 (28.3%)		196 (43.8%)	45 (10%)	
Rectum	169 (37.7%)	38 (8.5%)		133 (29.7%)	74 (16.5%)		151 (33.7%)	56 (12.5%)	
Tumor Size			0.001			0.005			0.064
<5	202 (45.1%)	48 (10.7%)		153 (34.2%)	97 (21.7%)		185 (41.3%)	65 (14.5%)	
≥5	133 (29.7%)	65 (14.5%)		94 (21%)	104 (23.2%)		162 (36.2%)	36 (8%)	
Stage			0.520			0.117			0.263
I+II	191 (42.6%)	69 (15.4%)		152 (33.9%)	108 (24.1%)		196 (43.8%)	64 (14.3%)	
III+IV	144 (32.1%)	44 (9.8%)		95 (21.2%)	93 (20.8%)		151 (33.7%)	37 (8.3%)	
Grade			0.202			0.988			0.905
G1+G2	316 (70.5%)	102 (22.8%)		231 (51.6%)	187 (41.7%)		323 (72.1%)	95 (21.2%)	
G3+M	19 (4.2%)	11 (2.5%)		16 (3.6%)	14 (3.1%)		24 (5.4%)	6 (1.3%)	
NVI			0.454			0.699			0.494
No	208 (46.4%)	65 (14.5%)		153 (34.2%)	120 (26.8%)		208 (46.4%)	65 (14.5%)	
Yes	127 (28.3%)	48 (10.7%)		94 (21%)	81 (18.1%)		139 (31%)	36 (8%)	
CEA			0.770			0.685			1.000
<6.5	241 (53.8%)	79 (17.6%)		174 (38.8%)	146 (32.6%)		248 (55.4%)	72 (16.1%)	
≥6.5	94 (21%)	34 (7.6%)		73 (16.3%)	55 (12.3%)		99 (22.1%)	29 (6.5%)	
CA19-9			0.682			0.468			0.420
<27	266 (59.4%)	87 (19.4%)		191 (42.6%)	162 (36.2%)		270 (60.3%)	83 (18.5%)	
≥27	69 (15.4%)	26 (5.8%)		56 (12.5%)	39 (8.7%)		77 (17.2%)	18 (4%)	
Chemotherapy			0.030			0.002			0.821
No	178 (39.7%)	46 (10.3%)		140 (31.2%)	84 (18.8%)		172 (38.4%)	52 (11.6%)	
Yes	157 (35%)	67 (15%)		107 (23.9%)	117 (26.1%)		175 (39.1%)	49 (10.9%)	

NLR, neutrophil-to-lymphocyte ratio; PLR, platelet-to-lymphocyte ratio; BMI, body mass index; M, Mucinous adenocarcinoma; NVI, nerve or vascular invasion; CEA, carcinoembryonic antigen; CA19-9, carbohydrate antigen 19–9.

### 3.2 NLR, PLR, and LMR cut-off value

**Figure 1:**
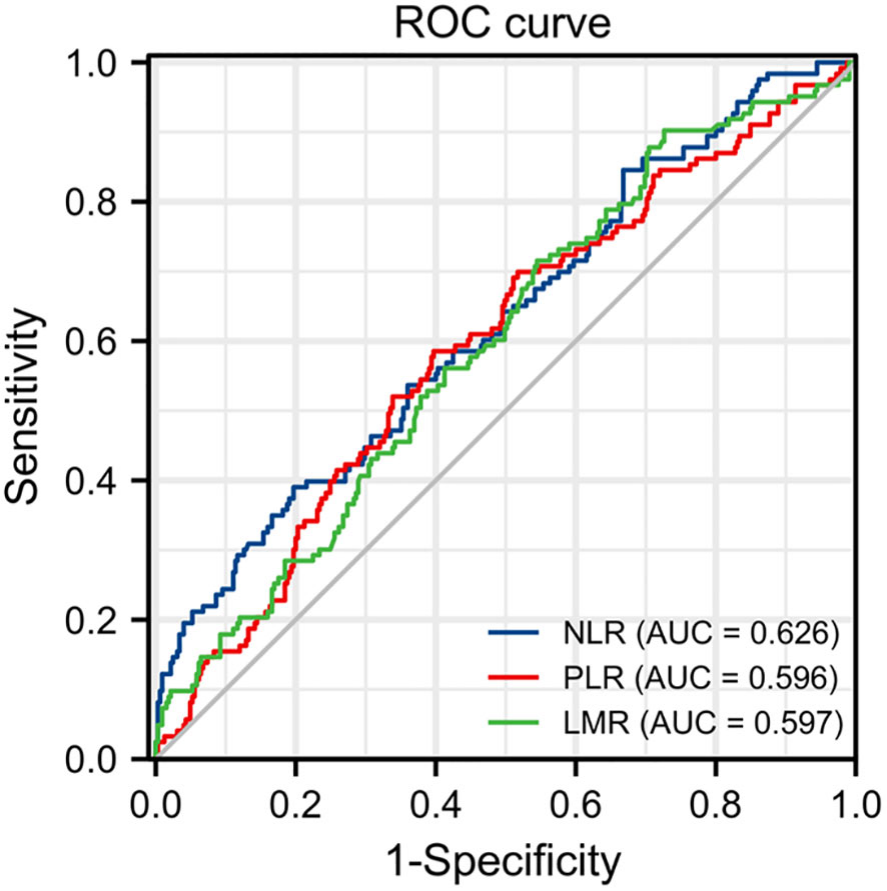
ROC curves of NLR, PLR and LMR in stage I-IV CRC.

The optimal cutoff points for NLR, PLR and LMR was calculate by the ROC curve. The optimal cut-off value of NLR was calculated as 2.81 with the areas under the curve (AUC) = 0.626, a sensitivity of 39.0%, a specificity of 80.3%; the PLR was 168.24 with AUC = 0.596, a sensitivity of 58.5%, a specificity of 60.3%; and the LMR was 5.46 with AUC = 0.597, a sensitivity of 90.2%, a specificity of 27.4%, by the AUC with the Youden index (**Figure 1**). All the patients were divided into high and low groups according to the cut-off values.

### 3.3 Kaplan–Meier survival analysis

**Figure 2:**
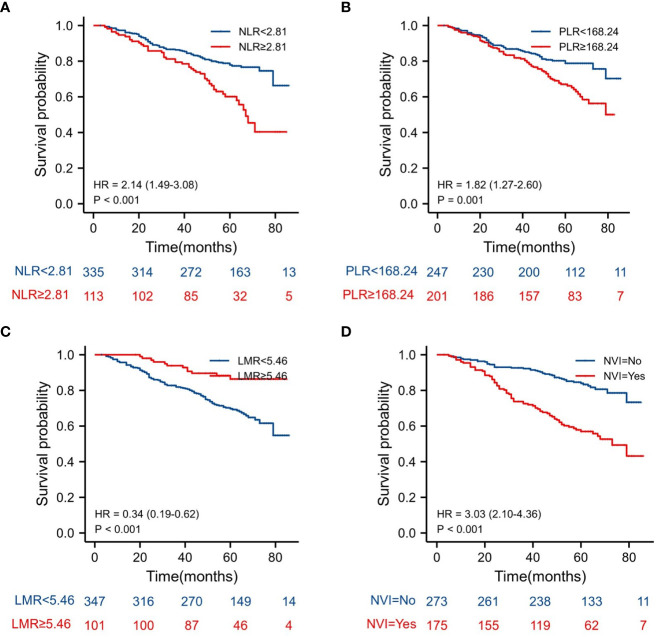
Kaplan–Meier estimates of overall survival (OS) according to the NLR, PLR, LMR and NVI in stage I-IV: **(A)** according to NLR (HR = 2.15, *P* < 0.001), **(B)** according to PLR (HR = 1.82, P = 0.001), **(C)** according to LMR (HR = 0.34, *P* < 0.001), and **(D)** according to NVI (HR = 3.03, P < 0.001).

**Figure 3:**
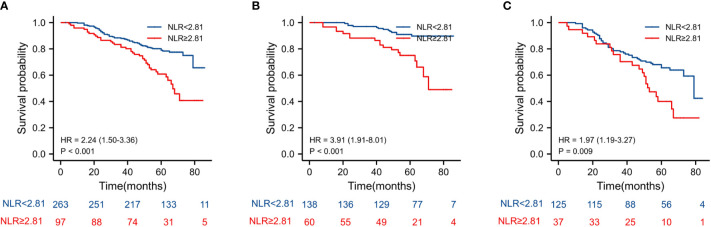
Kaplan–Meier estimates of overall survival (OS) according to the NLR in different stage group: **(A)** according to NLR in stage II-III (HR = 2.24, *P* < 0.001), **(B)** according to NLR in stage II (HR = 3.91, *P* < 0.001), and **(C)** according to NLR in stage III (HR = 1.97, *P* = 0.009).

**Figure 4:**
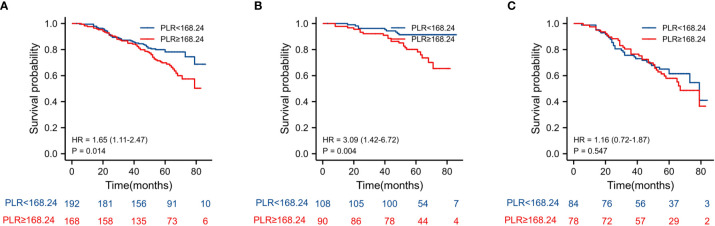
Kaplan–Meier estimates of overall survival (OS) according to the PLR in different stage group: **(A)** according to PLR in stage II-III (HR = 1.65, P =0.014), **(B)** according to PLR in stage II (HR = 3.09, P =0.004), and **(C)** according to PLR in stage III (HR = 1.16, P = 0.547).

**Figure 5:**
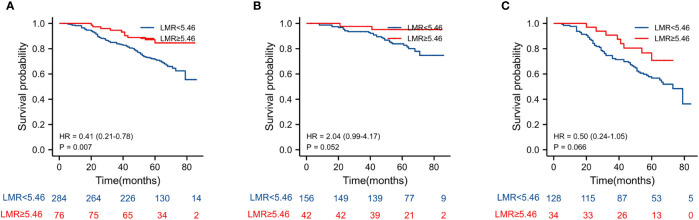
Kaplan–Meier estimates of overall survival (OS) according to the LMR in different stage groups: **(A)** according to LMR in stage II-III (HR = 0.41, *P* = 0.007), **(B)** according to LMR in stage II (HR = 2.04, *P* = 0.052), and **(C)** according to LMR in stage III (HR = 0.5, *P* = 0.066).

**Figure 6:**
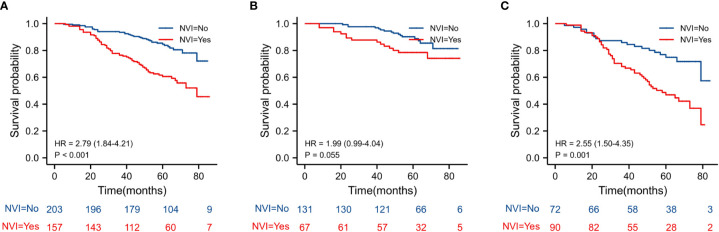
Kaplan–Meier estimates of overall survival (OS) according to the NVI in different stage groups: according to NVI in stage II-III (HR = 2.79, *P* < 0.001) according to NVI in stage II (HR = 1.99, *P* = 0.055) according to NVI in stage III (HR = 2.55, *P* = 0.001).

The Kaplan–Meier estimates of overall survival according to NLR, PLR, LMR, and NVI are shown in **Figures 2**–**6**. **Figure 2** shows that the 5-year OS rate was significantly lower in the high NLR or PLR group than in the low NLR or PLR group in postoperative stage I-IV CRC (*P* < 0.001; respectively, *P* = 0.001). High preoperative LMR indicated a higher 5-year OS rate in CRC patients (*P* < 0.001). Significant differences were also observed regarding OS among the NVI groups (*P* < 0.001). According to the subgroup analysis results, the NLR value rise in stage II-III CRC indicates a poor prognosis. The predictive value of prognosis in stage II CRC is better than in stage III CRC (HR = 3.91, *P* < 0.001 vs. HR = 1.97, *P* = 0.009) *(* **Figure 3**). For PLR groups, it is the similar (HR = 3.09, *P* = 0.004 vs. HR = 1.16, *P* = 0.547) (**Figure 4**). There was no difference in stage II and stage III between high and low LMR groups (HR = 0.24, *P* =0.052 vs. H R= 0.5, *P* = 0.066) (**Figure 5**). On the contrary, the NVI present in stage III CRC indicates a poor prognosis (HR = 1.99, *P* = 0.055 vs. HR = 2.55, *P* = 0.001) *(* **Figure 6**).

### 3.4 Univariate and multivariate analysis of overall survival

The univariate analysis of Cox proportional hazards reports showed that NLR and PLR are risk factors in terms of survival (HR = 2.140, 95% CI= (1.488-3.078), *P* < 0.001, respectively HR = 1.820, 95% CI = (1.271-2.605), *P* = 0.001), and LMR was a protective factor (HR = 0.341, 95% CI = (0.188-0.618), *P* < 0.001) (**Table 2**). Additionally, positive CEA/CA19-9, stage III+IV, NVI (+) and adjuvant chemotherapy have poorer overall survival in postoperative CRC patients. No significant interaction between survival and sex, age, BMI, tumor size, differentiation and site (**Tables 2**, **3**). From **Table 2**, **3**, we found in a univariate analysis that positive CEA/CA-199, stage III+IV, NVI (+) and adjuvant chemotherapy groups were an elevated risk of death in CRC patients after receiving operation. Multivariate analysis revealed that the increase of NLR and NVI (+) resulted in an increase in the risk of death (HR = 1.678, 95%CI = (1.114-2.527), *P* = 0.013), and for the LMR, a reduction of the risk of death (HR = 0.480, 95%CI = (0.256-0.902), *P* = 0.023) (**Table 3**). No difference was observed in PLR groups. These results suggest that NLR is an independent risk factor, and LMR is an independent protective factor in CRC patients after surgery.

**Table 2:** Univariate and Multivariate Analysis of Factors Associated with Overall Survival (n=448).

Characteristics	Total (N)	Univariate analysis		Multivariate analysis	
		Hazard ratio (95% CI)	P value	Hazard ratio (95% CI)	P value
Sex	448				
female	185	Reference			
male	263	1.128 (0.785-1.620)	0.516		
Age	448				
<60	234	Reference			
≥60	214	0.948 (0.665-1.352)	0.769		
BMI	448				
<25	332	Reference			
≥25	116	1.028 (0.684-1.545)	0.893		
Stage	448				
I+II	260	Reference			
III+IV	188	5.020 (3.353-7.516)	<0.001	3.904 (2.513-6.062)	<0.001
Grade	448				
G1+G2	418	Reference			
G3+M	30	2.753 (1.599-4.739)	<0.001	1.951 (1.120-3.399)	0.018
Size	448				
<5	250	Reference			
≥5	198	1.102 (0.773-1.572)	0.591		
Site	448				
Colon	241	Reference			
Rectum	207	0.834 (0.582-1.194)	0.321		
NVI	448				
No	273	Reference			
Yes	175	3.021 (2.099-4.349)	<0.001	1.999 (1.367-2.923)	<0.001
Chemotherapy	448				
No	224	Reference			
Yes	224	1.715 (1.188-2.476)	0.004	0.945 (0.645-1.383)	0.770

BMI, body mass index; M, Mucinous adenocarcinoma; NVI, nerve or vascular invasion.

**Table 3:** Univariate and Multivariate Analysis of Factors Associated with Overall Survival (n=448).

Characteristics	Total (N)	Univariate analysis		Multivariate analysis	
		Hazard ratio (95% CI)	P value	Hazard ratio (95% CI)	P value
CEA	448				
<6.5	320	Reference			
≥6.5	128	1.629 (1.132-2.344)	0.009	1.020 (0.688-1.512)	0.922
CA19-9	448				
<27	353	Reference			
≥27	95	1.904 (1.302-2.784)	<0.001	1.200 (0.794-1.814)	0.387
NLR	448				
NLR<2.81	335	Reference			
NLR≥2.81	113	2.140 (1.488-3.078)	<0.001	1.664 (1.105-2.508)	0.015
PLR	448				
PLR<168.24	247	Reference			
PLR≥168.24	201	1.820 (1.271-2.605)	0.001	1.111 (0.740-1.666)	0.612
LMR	448				
LMR<5.46	347	Reference			
LMR>5.46	101	0.341 (0.188-0.618)	<0.001	0.478 (0.255-0.898)	0.022

CEA, carcinoembryonic antigen; CA19-9, carbohydrate antigen 19–9; NLR, neutrophil-to-lymphocyte ratio; PLR, platelet-to-lymphocyte ratio; LMR, lymphocyte-to-monocyte ratio.

## 4 Discussion

Recently, numerous studies have confirmed the connection between human malignancy and systemic inflammatory processes. There is accumulating evidence that inflammatory biomarkers may affect a patient’s prognosis for malignancies. Inflammatory biomarkers have emerged into predictors of long-term overall survival across a number of malignancies. In the current research, we found that preoperative NLR, PLR, LMR, CEA, and CA19-9, as well as TNM stage and NVI, were significantly associated with overall survival in CRC patients. NLR is an independent risk factor in the multivariate regression analysis, whereas LMR is an independent protective factor in CRC patients following surgery.

Leukocytes, including neutrophils, monocytes, and lymphocytes, were engaged in systemic inflammatory responses in tumor patients, which have since grown into potential predictors of the clinical outcomes of various tumors (5, 7, 11). Neutrophils are the first responders to inflammation, infection, and injury. Neutrophil recruitment to the tumor microenvironment (TME) is mediated by various mediators, including cytokines, chemokines, lipids, and growth factors secreted from cancer cells and cancer-associated stromal cells. In turn, neutrophil promotes tumor cell proliferation and metastasis (12). Lymphocytes are part of the immune system that are two main types of lymphocytes: B cells and T cells. Lymphocytes are essential for immunological monitoring of malignancy. They prevent the proliferation and migration of tumor cells by generating cytotoxicity and cell death (13). Monocytes are innate immune cells of the mononuclear phagocyte system that exhibit diverse functions at different stages of tumor growth and progression (14). Growing evidence display a linkage between inflammation and cancer (15). These inflammatory response markers have become predictive factors for the prognosis.

Our study included 448 patients who underwent resection and prospective follow-up for at least 5 years and demonstrates that an NLR ≥ 2.81 is an independent risk factor for overall survival. Increased neutrophil-lymphocyte-ratio reflecting inflammatory responses *in vivo* represents tumor inflammatory status and a balance between protumor and antitumor immunity. Thus, an imbalance in the NLR has been closely associated with tumor progression and prognosis (11, 16). A retrospective clinical study initiated by Kubo et al. showed that a preoperative NLR > 2.1 was a poor tumor-specific survival predictive factor in CRC patients, particularly in advanced CRC patients (17). Similarly, increased preoperative NLR > 5 is associated with poorer long-term survival in patients with localized CRC and those with liver metastasis (18). Our results are consistent with other studies.

Tumor-associated proinflammatory cytokines also stimulate megakaryocytes and induce thrombocytosis. Platelets can promote the formation of tumor trophoblasts and increase micro-vessel permeability, facilitating tumor growth and metastasis (19). The platelet-lymphocyte-ratio (PLR) is closely related to the prognosis of CRC patients. Increased PLR was associated with shorter overall survival in patients with left-sided colon cancer and was more pronounced in patients with advanced TNM stage (20). In the present study, preoperative PLR ≥ 168.24 indicate a poor prognosis of stage I-IV CRC. However, preoperative PLR was not an independent risk factor for colorectal cancer. A retrospective study that included 391 complicated colorectal cancer showed that PLR is an independent risk factor in patients with complicated colorectal cancer (21). Derek J et al. found that preoperative PLR ≥ 220 was associated with poor overall survival in patients with resectable colorectal liver metastases (22). The current study displayed that preoperative PLR was not associated with survival in multivariate analysis, resulting from different inclusion criteria and patient characteristics.

Monocytes, the innate immune cells of the mononuclear phagocyte system, have emerged as important regulators of cancer development and progression. The value of the lymphocytes-to-monocytes ratio is a biomarker of the host’s immune response. Nishijima et al. found that the low preoperative value of LMR may be a poor and significant predictor of clinical outcomes in patients with colorectal cancer (23). A retrospective study including 87 patients revealed that left-sided colorectal cancer patients with high preoperative LMR had a longer five-year overall survival (24). In patients with locally advanced rectal cancer, elevated LMR was prominently correlated with worse prognostic features and an independent factor for better OS (25). In this study, we found that CRC patients with preoperative LMR ≥ 5.46 had an improved prognosis. Elevated LMR was found to be an independent prognostic factor of better OS by multivariate analysis. These results suggest that LMR is a useful prognostic tool for estimating the OS in colorectal cancer with several stages.

In stage I-IV CRC, other parameters such as clinical TNM stage, NVI, CEA, and CA19-9 are strongly related to long-term survival. Tumor markers are a common clinical tool in oncology in combination with other clinical and radiologic data. Tumor markers CEA and CA 19-9 have specific clinical applications for gastrointestinal cancer (26). These markers levels are significantly linked to colorectal cancer lesions. Patients with raised serum CEA and CA19-9 levels at diagnosis indicate a poor prognosis, resulting from lymph node metastasis and liver metastases. As a result, CRC patients with a high risk for death are identified by raised CEA and CA 19-9 levels, which can be utilized to select patients for adjuvant therapy (27). We reviewed clinical data from surgical patients over the last 5 years. We found that serum CEA and CA19-9 levels are prognostic factors for stage, metastases, and survival time in patients with CRC. Although elevated CEA and CA19-9 serum levels are independently predictive factors for advanced pancreatic cancer (28), they are not independent predictors in post-operation patients with CRC, according to our data. Compared with serum markers, pathological characteristics are the critical indicators for diagnosis and neoadjuvant therapy of CRC patients. Consistent with other studies, our results demonstrated that both TNM stage and NVI are independent prognostic factors in CRC patients after surgery.

The prognosis of different stages of CRC varies greatly, whereas early-onset CRC is insidious and failure to be diagnosed. NLR was reported to predict tumor staging in patients with colorectal cancer (29). The patient population enrolled in each study was different stage and treatment strategies, but most papers analyzed “pre-NLR” in patients with stage II-III colorectal cancer (7). High NLR (≥ 2.81) is an independent predictor for OS, according to our analysis of 448 Stage I–IV CRC patients who underwent radical resection. We then used Kaplan-Meier Survival Analysis to examine CRC patients with stages II-III. We discovered that stage II–III CRC patients with high NLR indexes had poor prognoses, but stage II CRC patients with NLR were better at predicting overall survival. We also analyzed PLR, LMR and NVI in stage II-III CRC patients. Interestingly, PLR was only associated with overall survival in stage II patients. Conversely, NVI was associated with the poor prognosis of stage III patients. Fu et al. analyzed 708 stage II CRC patients. They found that chemotherapy patients with high PLR had significantly longer overall survival and cancer-specific survival than non-chemotherapy patients (30). However, Davari et al. also analyzed 184 locally recurrent rectal cancer patients and found that NLR >3.9 and PLR >275 were related to a lower 5-yr overall survival (31). These findings suggest that the different predictive roles of PLR in CRC patients may have resulted from the stage, disease progression, and neoadjuvant chemotherapy. Surgery is often reserved for patients with stage II-III CRC in this single-center clinical study. Therefore, we focused on stage II-III CRC cancer and found different roles of NLR, PLR, and NVI in the prognosis. Our data demonstrated that NLR is an independent risk factor in colorectal cancer and is more suitable for predicting the prognosis of stage II CRC.

## 5 Conclusion

Prognostic scores based on inflammation, such as NLR, PLR, and LMR, results of the systemic inflammatory response, have been linked to survival in patients with colorectal cancer. The univariate analysis shows that the high values of NLR and PLR are risk factors, and the high value of LMR is a protective factor for the survival of postoperative patients with colorectal cancer. The increased value of NLR is an independent risk factor for colorectal cancer patients, while the increased value of LMR is a protective independent survival factor.

## Data availability statement

The original contributions presented in the study are included in the article/Supplementary Material. Further inquiries can be directed to the corresponding authors.

## Ethics statement

Written informed consent was obtained from the individual(s) for the publication of any potentially identifiable images or data included in this article.

## Author contributions

WD conceived the study. ZX and XC collected all colorectal cancer patients’ clinical records and follow-up data. XW conducted the Kaplan–Meier analysis. JWZ and HZ conducted the univariate and multivariate analysis. ZX wrote the manuscript. JNZ revised the manuscript for important intellectual content. WD supervised the study. All authors contributed to the article and approved the submitted version.
